# Insulin Glulisine Versus Regular Human Insulin for Prednisolone-Associated Hyperglycemia Assessed by Continuous Glucose Monitoring: An Open-Label Crossover Study

**DOI:** 10.7759/cureus.104228

**Published:** 2026-02-25

**Authors:** Hiroki Takizawa, Hirotsugu Uzawa, Masahiro Masuzawa, Osamu Ogawa

**Affiliations:** 1 Department of Diabetes and Endocrinology, Kameda Medical Center, Kamogawa, JPN; 2 Department of Metabolism and Endocrinology, Juntendo University Graduate School of Medicine, Tokyo, JPN; 3 Information Management Headquarters, Kameda Medical Center, Kamogawa, JPN

**Keywords:** continuous glucose monitoring, crossover study, insulin glulisine, regular human insulin, steroid-induced hyperglycemia

## Abstract

Background: Steroid (glucocorticoid)-induced hyperglycemia is common in hospitalized patients treated with prednisolone and often requires prandial insulin. Evidence is limited regarding whether rapid-acting insulin analogs provide different glycemic profiles compared with regular human insulin under protocol-based titration.

Methods: We conducted a single-center, non-randomized, open-label, crossover study in hospitalized adults with type 2 diabetes or steroid-induced diabetes who were receiving morning oral prednisolone (≥5 mg/day) and required insulin therapy. After an insulin dose-adjustment period (with non-insulin agents withheld throughout the dose-adjustment and study periods), participants received insulin glulisine and regular human insulin on consecutive study days without a washout interval, switching unit-for-unit at identical pre-meal doses. Glucose profiles were assessed using continuous glucose monitoring (CGM). Primary endpoints were total glucose area under the curve (AUC) over 0-24 hours and time-segment AUCs (0-8, 8-12, 12-18, and 18-24 hours).

Results: Thirteen of 26 eligible patients provided informed consent; six were excluded because >25% of expected CGM readings were missing, all due to device-related recording issues, leaving seven patients for analysis. The total AUC did not differ between insulin glulisine and regular human insulin (118.02 ± 30.95 vs 117.41 ± 30.20 ×10^2 mmol/L·min; P = 0.925), and no significant differences were observed in time-segment AUCs. Mean glucose, maximum glucose, minimum glucose, mean amplitude of glycemic excursions, and time-in-range metrics were similar. Coefficient of variation was lower with insulin glulisine (28.81 ± 9.05% vs 34.55 ± 8.96%; P = 0.034). In an exploratory post hoc analysis within a 30-minute time window around 16:00, AUC, mean glucose, and maximum glucose were lower with regular human insulin than with insulin glulisine (P < 0.05).

Conclusions: Under protocol-based titration during prednisolone treatment, insulin glulisine and regular human insulin produced comparable overall 24-hour glucose exposure. Findings on glycemic variability and post hoc time window analyses should be interpreted as exploratory and warrant confirmation in larger studies.

## Introduction

Glucocorticoid therapy remains widely used for the treatment of various autoimmune diseases. In population-based longitudinal cohorts from the United States, Taiwan, and Denmark, the average annual prevalence of systemic oral corticosteroid use during 2009-2018 was reported as 6.8%, 17.5%, and 2.2%, respectively [1]. In the inpatient setting, a UK single-center point-prevalence survey found that 12.8% of adult inpatients were receiving glucocorticoid therapy [2], and a large UK inpatient electronic health record cohort reported that 3.8% of inpatients without diabetes and not receiving glucocorticoids at admission newly initiated systemic glucocorticoid treatment during hospitalization, of whom 1.8% developed steroid (glucocorticoid)-induced hyperglycemia (SIH) [3]. In a meta-analysis of nondiabetic patients, SIH was estimated to develop in 32.3% after the initiation of glucocorticoid therapy [4]. In Japan, among hospitalized nondiabetic patients receiving glucocorticoids for rheumatic or renal diseases, 65.6% developed SIH during a four-week observation period [5]. Thus, SIH is common in inpatient settings and frequently necessitates insulin therapy for glycemic management.

SIH is characterized by prominent postprandial hyperglycemia even when fasting glucose levels remain within the normal range [6]. When basal insulin alone is insufficient, additional prandial (bolus) insulin is often required; however, evidence is limited regarding the optimal bolus insulin formulation for SIH. In particular, prednisolone, an intermediate-acting glucocorticoid, tends to cause hyperglycemia with marked diurnal variation, yet few studies have evaluated how different bolus insulin preparations influence glucose profiles in SIH.

Therefore, we aimed to compare 24-hour glucose profiles assessed by continuous glucose monitoring (CGM) on days of insulin glulisine (G) versus regular human insulin (R) in patients with type 2 diabetes or steroid-induced diabetes who required insulin therapy during prednisolone treatment.

## Materials and methods

Patients

This was a single-center, non-randomized, open-label, crossover study. We enrolled hospitalized patients at Kameda Medical Center between November 2012 and September 2013 who were receiving morning oral prednisolone (≥5 mg/day) and required insulin therapy for glycemic management. Eligible participants had type 2 diabetes or steroid-induced diabetes.

Prednisolone was required to be continued at a stable dose for at least one week as maintenance therapy; patients prescribed short-term prednisolone for conditions such as acute exacerbations of asthma or chronic obstructive pulmonary disease were excluded. We included only patients in whom no prednisolone dose changes were anticipated during the dose-adjustment period. Patients receiving enteral tube feeding or parenteral nutrition were not eligible; only those with stable oral intake of standard hospital meals were included.

Steroid-induced diabetes was defined as new-onset diabetes requiring insulin therapy during prednisolone treatment in patients without a prior history of diabetes.

Inclusion and exclusion criteria

Eligible participants were adults aged 20-80 years who had type 2 diabetes or steroid-induced diabetes and were hospitalized while receiving morning oral prednisolone at a dose of ≥5 mg/day. Prednisolone was required to be continued at the same dose for at least 1 week as maintenance therapy, and no dose changes were anticipated during the insulin dose-adjustment period. All participants had stable oral intake of standard hospital meals (i.e., no enteral tube feeding or parenteral nutrition). In addition, the attending physician determined that insulin therapy was necessary; as an operational criterion, participants required a total daily insulin dose of ≥10 units to achieve random whole-blood glucose values <11.1 mmol/L (200 mg/dL) measured by point-of-care testing (POCT).

Patients were excluded if they had type 1 diabetes; severe ketosis or diabetic coma within the preceding six months; pregnancy or possible pregnancy; renal dysfunction (estimated glomerular filtration rate <45 mL/min/1.73 m²); hepatic dysfunction (aspartate aminotransferase or alanine aminotransferase >3 times the upper limit of normal); current use of neutral protamine Hagedorn (NPH) insulin; ongoing enteral nutrition therapy; or inability to maintain adequate intake of hospital meals.

Study protocol

**Figure 1:**
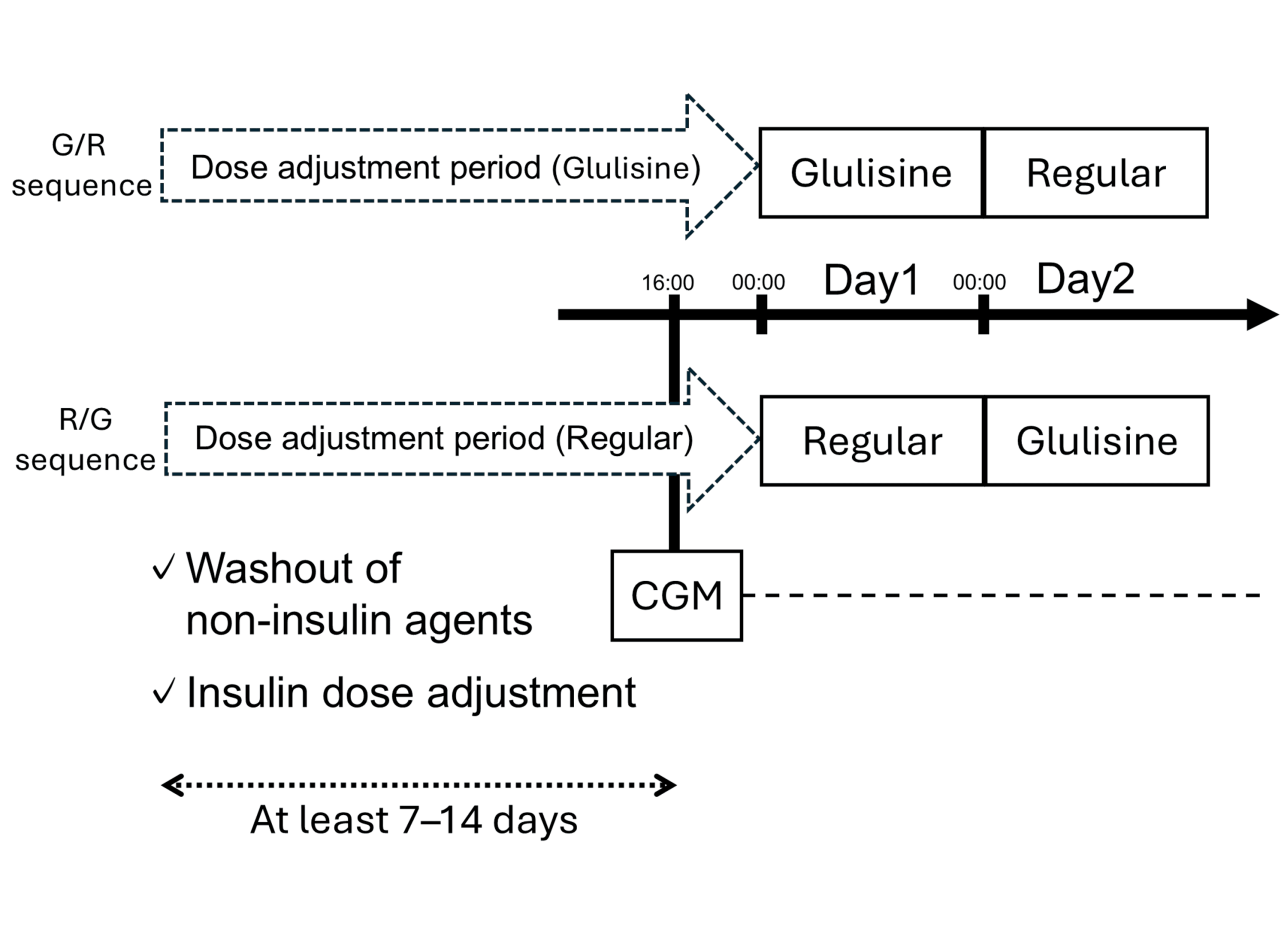
Study protocol and crossover design Participants received insulin glulisine and regular human insulin on consecutive study days in a crossover manner. CGM, continuous glucose monitoring; G, insulin glulisine; R, regular human insulin.

The study protocol is outlined in Figure 1. After informed consent was obtained, all non-insulin glucose-lowering medications (oral agents and, when applicable, glucagon-like peptide-1 receptor agonists) were withheld throughout the dose-adjustment and study periods. During the dose-adjustment period, basal insulin (insulin glargine U100), when used, was continued and adjusted as needed, and bolus insulin doses were titrated.

**Figure 2:**
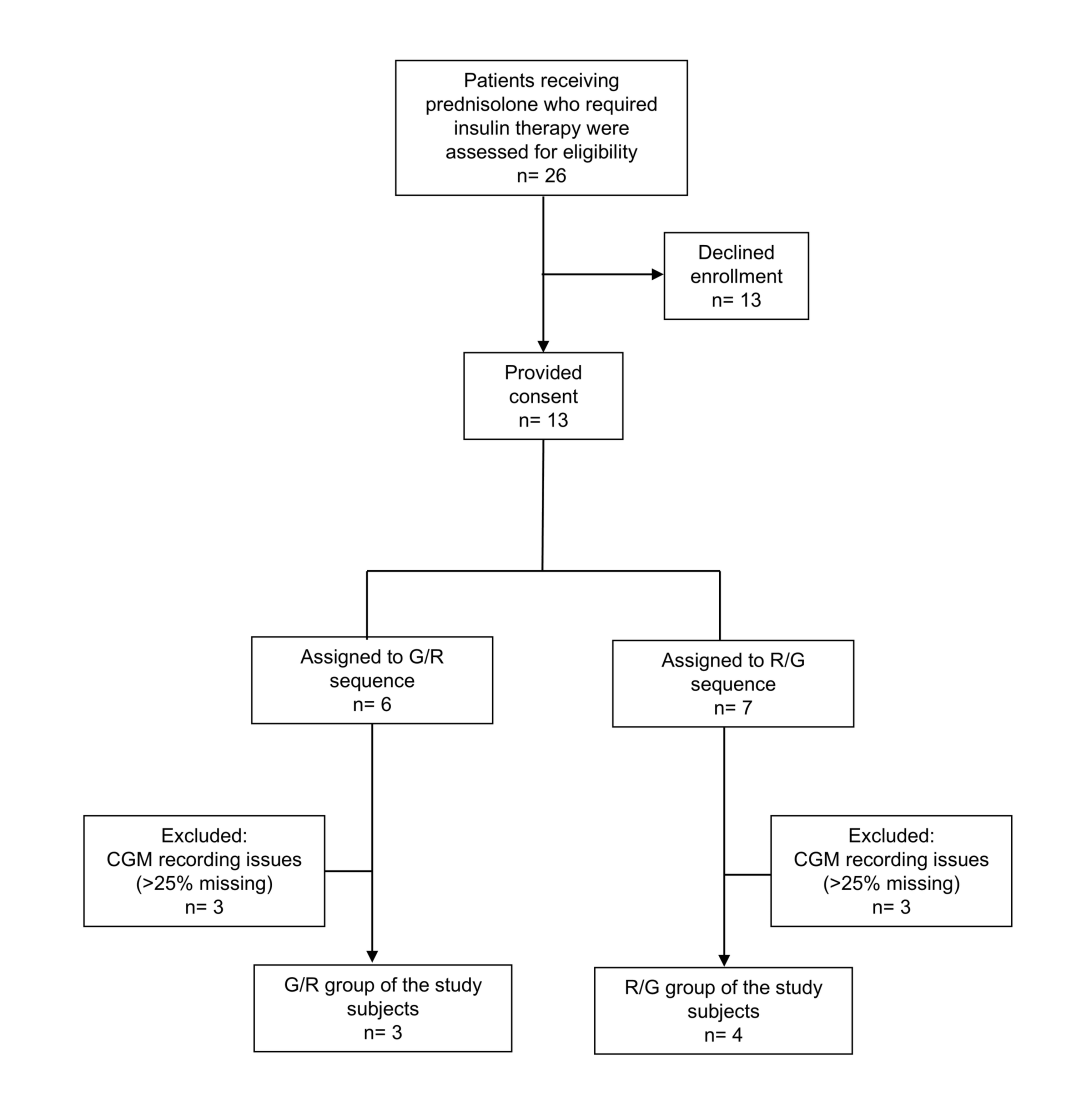
Participant flow diagram Participants were excluded if >25% of expected CGM readings were missing; all exclusions were due to device-related recording issues. CGM, continuous glucose monitoring; G, insulin glulisine; R, regular human insulin.

Thirteen hospitalized patients who required insulin therapy for glycemic management during prednisolone treatment and provided informed consent were enrolled. The enrollment and analysis flow is summarized in Figure 2. Patients were assigned alternately in order of enrollment to either the G/R sequence (insulin glulisine on Day 1 followed by regular human insulin on Day 2) or the R/G sequence (regular human insulin on Day 1 followed by insulin glulisine on Day 2). No washout interval between treatments was included; only the bolus insulin preparation was switched between Day 1 and Day 2 at the same pre-meal unit doses. Insulin glulisine was administered immediately before meals, whereas regular human insulin was administered 30 minutes before meals.

The dose-adjustment period lasted 7-14 days. Because the CGM device could record glucose for only three days, CGM was initiated at 16:00 on the day before Day 1. On Day 1 and Day 2, patients received the bolus insulin preparation specified by their assigned sequence; on Day 2, the bolus insulin was switched to the alternate preparation at the same pre-meal unit doses used on Day 1. CGM was removed on Day 3, and analyses were performed using CGM data from Day 1 and Day 2. For each study day, CGM metrics were calculated using data from 00:00 to 24:00 (midnight-to-midnight). Time-window analyses (0-8, 8-12, 12-18, and 18-24 hours) were defined accordingly.

Outcomes

The primary endpoints were the total area under the curve (AUC) for glucose over 0-24 hours and time-segment AUCs over 0-8, 8-12, 12-18, and 18-24 hours. The AUC was calculated by trapezoidal integration of CGM glucose values (mmol/L) obtained every 5 minutes and is presented as ×10^2 mmol/L·min.

Secondary endpoints included mean glucose (mean ± standard deviation), maximum glucose, and minimum glucose over 0-24 hours and within each time-segment; indices of glycemic variability (mean amplitude of glycemic excursions (MAGE), within-day standard deviation, and coefficient of variation (CV%)) over 0-24 hours; and CGM-derived clinical metrics, defined as time in range (TIR: 3.9-10.0 mmol/L (70-180 mg/dL)), time below range (TBR: <3.9 mmol/L (<70 mg/dL) and <3.0 mmol/L (<54 mg/dL)), and time above range (TAR: >10.0 mmol/L (>180 mg/dL)).

MAGE over 0-24 hours was calculated from five-minute CGM data using EasyGV (University of Oxford, Oxford, UK) [7] with default settings, in which excursions are defined as deviations exceeding 1 SD. When EasyGV returned “No Deviations outside 1 SD”, MAGE was recorded as 0 mmol/L.

Total postprandial iAUC (8-24 hours) was calculated as the sum of meal-specific incremental AUCs (8-12, 12-18, and 18-24 hours) derived from five-minute CGM data after subtracting the pre-meal baseline glucose for each meal (without truncating negative values).

Insulin dose adjustments

During the insulin dose-adjustment period, basal insulin (insulin glargine U100), when used, was continued and adjusted as needed, and the assigned bolus insulin (insulin glulisine or regular human insulin), administered three times daily, was titrated to achieve fasting and pre-meal glucose levels of approximately 7.7 mmol/L (140 mg/dL), as measured by POCT whole-blood glucose testing.

Glucose monitoring

During the dose-adjustment period, point-of-care whole-blood glucose testing was performed using the Precision Xceed® system (Abbott Japan, Tokyo, Japan). During the study period, glucose profiles were assessed using a continuous glucose monitoring (CGM) system (CGMS® System Gold™, Medtronic MiniMed, Northridge, CA, USA). For CGM analyses, participants were excluded if >25% of expected CGM readings were missing. All exclusions were due to device-related recording issues.

Sample size estimation

Because any between-treatment difference was anticipated to be most evident during the post-breakfast period, the sample size was estimated based on the 8-12 hours AUC (a four-hour window). We assumed a clinically meaningful between-treatment difference in mean glucose of 2.0 mmol/L over four hours, corresponding to ΔAUC = 4.8 × 10^2 mmol/L·min. Variance assumptions were informed by a crossover study reporting glucose AUC (0-4 hours) as mean ± SEM [8]; the SEM was converted to SD, and the SD of paired differences was estimated assuming a moderate within-subject correlation (ρ = 0.5). A power analysis using G*Power indicated that 10 participants would be required to achieve 80% power at a two-sided α of 0.05 using a paired t-test [9]. To allow for potential attrition or missing CGM data, we aimed to enroll 12 participants.

Statistical analysis

Data are presented as mean ± SD or median (interquartile range), as appropriate. The primary endpoints (total AUC over 0-24 hours and time-segment AUCs over 0-8, 8-12, 12-18, and 18-24 hours) and secondary endpoints were compared between treatments (insulin glulisine vs regular human insulin) using paired t-tests. A two-sided P-value <0.05 was considered statistically significant. Statistical analyses were performed using IBM SPSS Statistics for Windows, Version 29 (Released 2023; IBM Corp., Armonk, New York, United States).

As a sensitivity analysis, the primary endpoints (total AUC and time-segment AUCs) were re-evaluated using a linear mixed-effects model with treatment (insulin glulisine vs regular human insulin), period (Day 1 vs Day 2), and sequence (G/R vs R/G) as fixed effects and a random intercept for participant.

## Results

Participants and treatment characteristics

Of 26 eligible patients, 13 provided informed consent. Of these 13 patients, six patients were excluded from the analysis because sufficient CGM data were not obtained (i.e., >25% of expected CGM readings were missing), all due to device-related recording issues, leaving seven patients for the final analysis. Baseline clinical characteristics and insulin treatment parameters of the analyzed patients are summarized in Table 1.

**Table 1:** Baseline characteristics and insulin treatment parameters before and after insulin adjustment Data are presented as median (interquartile range) unless otherwise indicated. Basal insulin use and total daily insulin dose are shown both before and after insulin adjustment. Total daily insulin dose was defined as the sum of basal and prandial insulin doses. BMI, body mass index; HbA1c, hemoglobin A1c; eGFR, estimated glomerular filtration rate; AST, aspartate aminotransferase; ALT, alanine aminotransferase.

Characteristic	Overall (n=7)
Male sex, n (%)	5 (71%)
Age (years)	62.0 (57.0-63.5)
BMI (kg/m^2^)	22.5 (19.3-24.1)
HbA1c (%)	7.2 (6.7-7.9)
Prednisolone (mg/day)	45 (25-50)
Serum creatinine (µmol/L)	75.1 (55.7-78.7)
eGFR (mL/min/1.73 m^2^)	72.6 (66.7-86.0)
AST (IU/L)	28.0 (17.5-32.5)
ALT (IU/L)	36.0 (23.5-46.5)
Use of oral hypoglycemic agents, n (%)	2 (29%)
Basal insulin use (insulin glargine U100), n (%)	
Before insulin adjustment	4 (57%)
After insulin adjustment	3 (43%)
Total daily insulin dose (U/day)	
Before insulin adjustment	34.0 (24.0-44.0)
After insulin adjustment	40.0 (34.0-44.0)

The prednisolone dose among the analyzed patients ranged from 7 to 100 mg/day. All seven patients were receiving insulin therapy at enrollment. Two patients were receiving sitagliptin 50 mg, which was discontinued according to the study protocol. Four patients were treated with insulin glargine (U100); during dose adjustment, insulin glargine was discontinued in one patient.

After bolus insulin titration, the pre-meal bolus insulin doses administered before breakfast, lunch, and dinner were 12.0 (11.0-14.0), 12.0 (10.0-13.0), and 5.0 (4.0-6.0) units, respectively. The total daily insulin dose was 40.0 (34.0-44.0).

Primary endpoint: AUC

The total AUC over 0-24 hours (×10^2 mmol/L·min) was 118.02 ± 30.95 with insulin glulisine and 117.41 ± 30.20 with regular human insulin, with no significant difference between treatments (P = 0.925). Likewise, there were no significant differences in time-segment AUCs between treatments (0-8 hours: 30.96 ± 10.39 vs 26.02 ± 10.12, P = 0.114; 8-12 hours: 18.91 ± 4.44 vs 20.73 ± 6.44, P = 0.291; 12-18 hours: 34.42 ± 11.19 vs 32.50 ± 9.63, P = 0.291; and 18-24 hours: 32.59 ± 12.74 vs 37.05 ± 9.91, P = 0.243) (Table 2).

**Table 2:** Comparison of CGM-derived glucose metrics between insulin glulisine and regular human insulin Data are presented as mean ± SD. P-values were calculated using a two-sided paired t-test, and the t statistic is reported as t (df = 6). Primary endpoints were BG-AUC (0-24 hours) and BG-AUC in each time window (0-8, 8-12, 12-18, and 18-24 hours). Total postprandial iAUC (8-24 hours) was calculated as the sum of meal-specific incremental AUCs (8-12, 12-18, and 18-24 hours) derived from five-minute CGM data after subtracting the pre-meal baseline glucose for each meal (without truncating negative values). MAGE was calculated using EasyGV from five-minute CGM data over 0-24 hours. When EasyGV returned “No Deviations outside 1 SD”, MAGE was recorded as 0 mmol/L. SD and CV were calculated using CGM data over 0-24 hours. No adjustment was made for multiple comparisons. BG, blood glucose; AUC, area under the curve; iAUC, incremental area under the curve; MAGE, mean amplitude of glycemic excursions; SD, standard deviation; CV, coefficient of variation; CGM, continuous glucose monitoring.

Parameter	Insulin glulisine day	Regular human insulin day	t (6)	P
BG-AUC (0-24 h), ×10^2 mmol/L·min	118.02 ± 30.95	117.41 ± 30.20	0.098	0.925
BG-AUC (0-8 h), ×10^2 mmol/L·min	30.96 ± 10.39	26.02 ± 10.12	1.851	0.114
BG-AUC (8-12 h), ×10^2 mmol/L·min	18.91 ± 4.44	20.73 ± 6.44	-1.159	0.291
BG-AUC (12-18 h), ×10^2 mmol/L·min	34.42 ± 11.19	32.50 ± 9.63	1.158	0.291
BG-AUC (18-24 h), ×10^2 mmol/L·min	32.59 ± 12.74	37.05 ± 9.91	-1.294	0.243
Total postprandial iAUC (8-24 h), ×10^2 mmol/L·min	12.15 ± 8.41	17.13 ± 10.22	-1.826	0.118
BG mean (0-24 h), mmol/L	8.22 ± 2.16	8.18 ± 2.10	0.106	0.919
BG mean (0-8 h), mmol/L	6.52 ± 2.18	5.48 ± 2.12	1.852	0.113
BG mean (8-12 h), mmol/L	8.01 ± 1.86	8.78 ± 2.71	-1.157	0.291
BG mean (12-18 h), mmol/L	9.68 ± 3.14	9.14 ± 2.70	1.148	0.294
BG mean (18-24 h), mmol/L	9.18 ± 3.58	10.41 ± 2.78	-1.280	0.248
BG max (0-24 h), mmol/L	14.08 ± 4.57	14.79 ± 3.19	-0.947	0.380
BG min (0-24 h), mmol/L	3.80 ± 0.93	3.69 ± 1.04	0.356	0.734
MAGE (0-24 h), mmol/L	3.88 ± 2.60	6.17 ± 1.77	-1.936	0.101
SD (0-24 h), mmol/L	2.46 ± 1.23	2.79 ± 0.95	-2.186	0.072
CV (0-24 h), %	28.81 ± 9.05	34.55 ± 8.96	-2.729	0.034

In the sensitivity analysis using a linear mixed-effects model, the treatment effect was not significant for the total AUC or for time-segment AUCs (total AUC: F(1,7) = 0.020, P = 0.890; 0-8 h: F(1,7) = 3.825, P = 0.091; 8-12 h: F(1,7) = 2.498, P = 0.158; 12-18 h: F(1,7) = 1.487, P = 0.262; and 18-24 h: F(1,7) = 2.626, P = 0.149). The treatment effect for the 0-8 hours AUC was not statistically significant, and the primary paired t-test analysis was also not significant (P = 0.114).

Secondary endpoints and exploratory analyses

No statistically significant differences were observed between treatments in mean glucose, maximum glucose, minimum glucose, or MAGE; MAGE was 3.88 ± 2.60 vs 6.17 ± 1.77 mmol/L (P = 0.101) (Table 2). Total postprandial iAUC (8-24 hours) tended to be lower with insulin glulisine, but the difference did not reach statistical significance (P = 0.118; Table 2).

Within-day standard deviation tended to be lower with insulin glulisine (2.46 ± 1.23 vs 2.79 ± 0.95 mmol/L, P = 0.072), and CV% was significantly lower with insulin glulisine (28.81 ± 9.05% vs 34.55 ± 8.96%, P = 0.034). Given the small sample size and the evaluation of multiple metrics, these findings should be interpreted as exploratory.

As an exploratory analysis, we calculated and compared time in range (TIR: 3.9-10.0 mmol/L), time below range (TBR: <3.9 mmol/L and <3.0 mmol/L), and time above range (TAR: >10.0 mmol/L); no statistically significant differences were observed between treatments for TIR, TBR, or TAR (Table 3).

**Table 3:** Exploratory CGM clinical metrics with insulin glulisine versus regular human insulin Data are presented as mean ± SD. P-values were calculated using a two-sided paired t-test, and the t statistic is reported as t (df = 6). No adjustment was made for multiple comparisons. TAR, time above range; TBR, time below range; TIR, time in range; CGM, continuous glucose monitoring

Parameter	Insulin glulisine day	Regular human insulin day	t (6)	P
TIR (3.9-10.0 mmol/L), %	72.22 ± 22.33	65.08 ± 18.97	1.824	0.118
TBR (<3.9 mmol/L), %	4.07 ± 5.43	6.85 ± 12.23	-0.973	0.368
TBR (<3.0 mmol/L), %	0.40 ± 1.05	5.56 ± 10.64	-1.307	0.239
TAR (>10.0 mmol/L), %	23.71 ± 22.23	28.08 ± 20.21	-1.232	0.264

**Figure 3:**
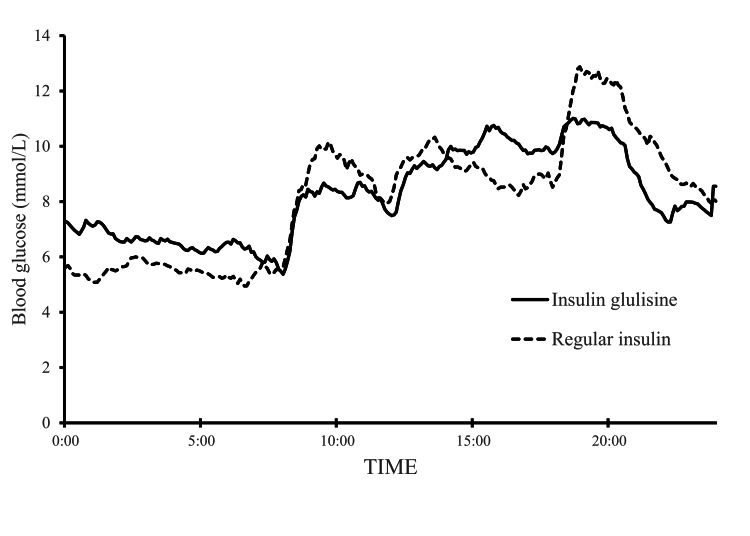
Mean 24-hour CGM glucose profiles Mean CGM glucose profiles during treatment with insulin glulisine (solid line) and regular human insulin (dashed line) are shown. Each curve represents the mean CGM glucose across all participants for each treatment day, regardless of treatment sequence, plotted at five-minute intervals over 00:00-24:00. Error bars are omitted for clarity. CGM, continuous glucose monitoring.

Figure 3 shows the mean 24-hour CGM glucose profiles averaged across all analyzed participants for insulin glulisine and regular human insulin.

As an exploratory analysis, we further compared AUC, mean glucose, maximum glucose, and minimum glucose within 30-minute windows (±15 minutes) centered at 10:00, 14:00, 16:00, and 20:00, where between-treatment differences appeared largest on visual inspection of Figure 3 (Table 4). The AUC for this ±15-minute window represents the absolute AUC over the 30-minute period without baseline subtraction and was not an iAUC measure.

**Table 4:** Exploratory glucose parameters within ±15 minutes of selected clock times with insulin glulisine versus regular human insulin Data are presented as mean ± SD. Each time window represents a 30-minute interval (±15 minutes) around the specified clock time. BG-AUC was calculated from five-minute CGM values using the trapezoidal rule. Mean, maximum, and minimum blood glucose (BG) values were derived from CGM data within each window. P-values were calculated using a two-sided paired t-test, and the t statistic is reported as t (df = 6). This was an exploratory, post hoc analysis (selected clock times were chosen based on visual inspection of the 24-hour CGM profile), and no adjustment was made for multiple comparisons. BG, blood glucose; AUC, area under the curve; CGM, continuous glucose monitoring.

Time window (±15 min)	Parameter	Insulin glulisine day	Regular human insulin day	t (6)	P
10:00	BG-AUC	2.51 ± 0.91	2.91 ± 1.07	-1.113	0.308
	Mean BG	8.37 ± 3.04	9.71 ± 3.60	-1.119	0.306
	Max BG	8.86 ± 3.03	10.34 ± 3.58	-1.187	0.280
	Min BG	7.87 ± 2.96	9.19 ± 3.76	-1.063	0.328
14:00	BG-AUC	2.88 ± 1.18	2.90 ± 1.11	-0.112	0.914
	Mean BG	9.61 ± 3.94	9.68 ± 3.73	-0.124	0.905
	Max BG	10.21 ± 4.32	10.32 ± 3.65	-0.163	0.876
	Min BG	8.97 ± 3.62	9.10 ± 3.85	-0.191	0.855
16:00	BG-AUC	3.16 ± 1.09	2.56 ± 0.65	2.654	0.038
	Mean BG	10.55 ± 3.66	8.56 ± 2.18	2.622	0.039
	Max BG	11.07 ± 3.70	9.07 ± 2.17	2.519	0.045
	Min BG	10.03 ± 3.66	8.18 ± 2.21	2.430	0.051
20:00	BG-AUC	3.18 ± 1.14	3.70 ± 1.07	-1.711	0.138
	Mean BG	10.59 ± 3.80	12.33 ± 3.59	-1.735	0.133
	Max BG	11.09 ± 3.85	12.95 ± 3.63	-2.051	0.086
	Min BG	10.05 ± 3.79	11.80 ± 3.67	-1.715	0.137

Around 16:00, values were lower with regular human insulin than with insulin glulisine for AUC (insulin glulisine vs regular human insulin: 3.16 ± 1.09 vs 2.56 ± 0.65 ×10^2 mmol/L·min, P = 0.038), mean glucose (10.55 ± 3.66 vs 8.56 ± 2.18 mmol/L, P = 0.039), and maximum glucose (11.07 ± 3.70 vs 9.07 ± 2.17 mmol/L, P = 0.045). By contrast, around 20:00, maximum glucose tended to be higher with regular human insulin, although the difference was not statistically significant (P = 0.086). As these analyses involved post hoc selection of multiple time points and outcomes and no adjustment for multiple comparisons, the results should be interpreted as exploratory.

## Discussion

This single-center, open-label, crossover study compared CGM-derived glucose metrics between insulin glulisine and regular human insulin in patients with type 2 diabetes or steroid-induced diabetes requiring insulin during prednisolone treatment. While prior work in glucocorticoid-induced hyperglycemia has primarily focused on basal insulin strategies (e.g., NPH insulin and insulin glargine), evidence regarding the optimal prandial insulin choice, particularly when assessed by CGM over 24 hours, remains limited. 

In the present study, no statistically significant differences were observed between insulin glulisine and regular human insulin in the primary endpoints, namely, total AUC (0-24 hours) and time-segment AUCs (0-8, 8-12, 12-18, and 18-24 hours). These findings suggest that, under our dose-adjustment protocol, the two bolus insulin preparations may yield comparable overall glucose exposure. The sensitivity analysis using a linear mixed-effects model was consistent with the primary analysis, with treatment effects remaining non-significant after accounting for period and sequence effects.

Similarly, no statistically significant differences were observed in secondary endpoints, including mean, maximum, and minimum glucose, and MAGE, suggesting comparable glycemic control between the two preparations under protocol-based titration. Glucocorticoids exacerbate postprandial hyperglycemia through mechanisms including increased insulin resistance [6,10,11]. During prednisolone treatment, fasting glucose is typically normal to mildly elevated, with hyperglycemia emerging from the afternoon to evening [12]. Although inpatient evidence for SIH management has largely focused on basal insulin-based strategies, direct comparative data on prandial insulin formulations assessed by 24-hour CGM during prednisolone treatment remain limited. We therefore used CGM to examine whether the choice of prandial insulin influences glucose profiles during these clinically relevant time periods.

In exploratory analyses, CV% was lower with insulin glulisine. Although mean glucose was similar between treatments, insulin glulisine may potentially attenuate glycemic variability. However, given the limited sample size and the evaluation of multiple metrics, this finding should be interpreted cautiously as exploratory.

Glucocorticoid therapy in hospitalized patients has been associated with dose-dependent increases in insulin requirements [13]. Long-acting glucocorticoids, such as dexamethasone, may induce sustained hyperglycemia that necessitates basal insulin therapy. The ADA Standards of Care in Diabetes-2026 state that NPH insulin may be considered as an option [14]. In the case of intermediate-acting glucocorticoids such as prednisolone, this approach is partly based on the alignment between the glycemic effect profile of intermediate-acting glucocorticoids and the pharmacodynamic profile of NPH insulin, which typically peaks around 4-6 hours after administration [15].

Although direct comparisons of insulin regimens remain limited, several studies have examined basal insulin strategies for SIH. In a retrospective study by Dhital et al., NPH insulin was associated with an approximately 20% lower total insulin dose than insulin glargine (U100) to achieve similar glycemic control in patients with type 2 diabetes [16]. In an open-label randomized trial, Grommesh et al. compared NPH plus insulin lispro with insulin glargine (U100) plus insulin lispro in 61 patients with SIH and reported lower glucose levels on Day 3 in the NPH group. However, interpretation requires caution because the glucocorticoid type was not standardized, and the NPH group received a 16% higher insulin dose [17]. Ruiz de Adana et al. found no significant differences in mean glucose or measures of glucose variability between NPH plus insulin glulisine and insulin glargine (U100) plus insulin glulisine in patients with type 2 diabetes receiving methylprednisolone or deflazacort [18]. Similarly, Radhakutty et al. reported comparable glucose outcomes when insulin doses were standardized between NPH plus insulin aspart and insulin glargine (U100) plus insulin aspart in patients receiving prednisolone at ≥20 mg/day [19].

Compared with rapid-acting insulin analogs such as insulin glulisine, regular human insulin has a slower onset and later peak (approximately 2 vs 1 hour), which may influence the optimal timing of administration and postprandial glucose excursions [8]. In a post hoc analysis within a 30-minute time window around 16:00, AUC, mean glucose, and maximum glucose were lower with regular human insulin than with insulin glulisine (P < 0.05). This could reflect the longer duration of action of regular human insulin, whereby the effect of the pre-lunch dose could persist into the late afternoon. Because afternoon-to-evening hyperglycemia is a typical pattern under intermediate-acting glucocorticoids, the observation around 16:00 is consistent with this clinical time window.

From a practical perspective, regular human insulin may offer potential benefit in stabilizing glucose around the late afternoon; however, this must be balanced against other considerations, including the lower CV% observed with insulin glulisine, the inconvenience of administering regular human insulin 30 minutes before meals, and the need to blunt early postprandial rises. One potential approach could be to use insulin glulisine for breakfast and dinner and regular human insulin at lunch. Nevertheless, given regimen complexity and injection burden, an initial strategy using NPH insulin may be considered, with subsequent adjustment using rapid-acting insulin analogs such as insulin glulisine if postprandial hyperglycemia remains prominent. Further studies are needed to clarify optimal strategies.

This study has several limitations. First, the analyzable sample size was smaller than anticipated because of CGM-related technical issues, primarily missing data. The CGM system used at the time (Medtronic MiniMed CGMS® System Gold™) was an early-generation retrospective CGM device that provided up to 72 hours of monitoring, and incomplete recordings have been reported with this platform. For example, in a study of 78 adults monitored with a Medtronic MiniMed CGMS® Gold device, eight CGM traces were excluded because of inadequate data [7], and in an adolescent sports-camp study, only 66% of sensors provided adequate signals for 48 hours [20]. Therefore, given the reduced analyzable sample size, the absence of statistically significant differences does not exclude the possibility of clinically meaningful between-treatment differences. Second, this was a single-center study, and heterogeneity in patient characteristics and glucocorticoid treatment conditions (type, dose, and timing) could not be fully accounted for. Third, with a small sample size (n = 7) and multiple endpoints assessed, the findings should be regarded as hypothesis-generating. In addition, the time window analyses were exploratory and did not include adjustment for multiple comparisons, warranting cautious interpretation and limiting generalizability.

## Conclusions

In conclusion, no statistically significant differences were observed between insulin glulisine and regular human insulin in the total AUC (0-24 hours) or time-segment AUCs under a protocol-based dose-adjustment approach, suggesting that either preparation may be a reasonable prandial option during prednisolone treatment when carefully titrated. Exploratory findings regarding glycemic variability (CV%) and time window analyses warrant confirmation in larger studies.
